# Her2 oncogene transformation enhances 5-aminolevulinic acid-mediated protoporphyrin IX production and photodynamic therapy response

**DOI:** 10.18632/oncotarget.11058

**Published:** 2016-08-04

**Authors:** Xue Yang, Pratheeba Palasuberniam, Kenneth A. Myers, Chenguang Wang, Bin Chen

**Affiliations:** ^1^ Department of Pharmaceutical Sciences, Philadelphia College of Pharmacy, University of The Sciences, Philadelphia, Pennsylvania, USA; ^2^ Department of Biological Sciences, Misher College of Arts and Sciences, University of The Sciences, Philadelphia, Pennsylvania, USA; ^3^ Key Laboratory of Tianjin Radiation and Molecular Nuclear Medicine, Institute of Radiation Medicine, Peking Union Medical College and Chinese Academy of Medical Sciences, Tianjin, China

**Keywords:** human epidermal growth receptor 2 (Her2), aminolevulinic acid (ALA), protoporphyrin IX (PpIX), photodynamic therapy (PDT), heme biosynthesis

## Abstract

Enhanced protoporphyrin IX (PpIX) production in tumors derived from the administration of 5-aminolevulinic acid (ALA) enables the use of ALA as a prodrug for photodynamic therapy (PDT) and fluorescence-guided tumor resection. Although ALA has been successfully used in the clinic, the mechanism underlying enhanced ALA-induced PpIX production in tumors is not well understood. Human epidermal growth receptor 2 (Her2, Neu, ErbB2) is a driver oncogene in human cancers, particularly breast cancers. Here we showed that, in addition to activating Her2/Neu cell signaling, inducing epithelial-mesenchymal transition and upregulating glycolytic enzymes, transfection of NeuT (a mutated Her2/Neu) oncogene in MCF10A human breast epithelial cells significantly enhanced ALA-induced PpIX fluorescence by elevating some enzymes involved in PpIX biosynthesis. Furthermore, NeuT-transformed and vector control cells exhibited drastic differences in the intracellular localization of PpIX, either produced endogenously from ALA or applied exogenously. In vector control cells, PpIX displayed a cell contact-dependent membrane localization at high cell densities and increased mitochondrial localization at low cell densities. In contrast, no predominant membrane localization of PpIX was observed in NeuT cells and ALA-induced PpIX showed a consistent mitochondrial localization regardless of cell density. PDT with ALA caused significantly more decrease in cell viability in NeuT cells than in vector cells. Our data demonstrate that NeuT oncogene transformation enhanced ALA-induced PpIX production and altered PpIX intracellular localization, rendering NeuT-transformed cells increased response to ALA-mediated PDT. These results support the use of ALA for imaging and photodynamic targeting Her2/Neu-positive tumors.

## INTRODUCTION

Photodynamic therapy (PDT) is a FDA-approved treatment modality using photoactivatable drugs called photosensitizers to generate therapeutic effects through the production of reactive oxygen species following light activation [1]. One PDT agent that has been used in the clinic is the aminolevulinic acid (ALA). As a prodrug, ALA itself does not possess any photosensitizing activity. It is metabolically converted in the heme biosynthesis pathway to protoporphyrin IX (PpIX), a heme precursor metabolite with photosensitizing and fluorescent properties [2, 3]. Heme biosynthesis pathway is a ubiquitous cell metabolic pathway existing in almost all mammalian cells. It begins with the formation of ALA in mitochondria, continues with consecutive enzymatic cascades in the cytosol, and ends with the chelation of PpIX and ferrous iron to form heme back in mitochondria. Administration of exogenous ALA bypasses the step of endogenous ALA synthesis where pathway negative feedback control is located, resulting in increased PpIX production [2, 3].

Because tumor tissues are often found to exhibit higher ALA-induced PpIX level than normal tissues, ALA has been used as a therapeutic agent for tumor treatment by PDT and imaging probe for fluorescence-guided tumor diagnosis and resection [1, 4]. PDT with ALA is widely used for the treatment of skin lesions such as actinic keratosis (AK) and basal cell carcinoma (BCC) with high efficacy and excellent cosmetic outcome [5, 6]. ALA-induced PpIX fluorescence is commonly used for guiding the resection of gliomas with improved resection rate and increased progression-free survival [7, 8]. Increase in overall survival was shown in a small cohort of glioblastoma patients treated with ALA and Photofrin-induced fluorescence-guided resection followed by repetitive PDT [9]. In addition to these clinical successes, ALA is also being actively explored as a tumor diagnostic and treatment agent for other types of cancers. For instance, higher ALA-induced PpIX fluorescence was detected in some human breast cancer cells than normal breast epithelial cells [10, 11]. Enhanced PpIX fluorescence in breast tumor cells led to the use of ALA for detecting mouse mammary tumors [12], and human primary breast tumors and lymph node metastases [13, 14]. Preferential ALA-induced PpIX production also enabled the use of PDT to inhibit breast cancer cell survival and tumor growth in animals [11, 15, 16].

Despite these promising results, the underlying mechanism involved in increased ALA-induced PpIX in tumors remains elusive [17]. Previous studies suggest that this is related to alterations in the expression and activity of heme biosynthesis enzymes including porphobilinogen deaminase (PBGD) and ferrochelatase (FECH) [18–21], and porphyrin transporters such as ATP-binding cassette family protein B6 (ABCB6) [22, 23]. Furthermore, pharmacological upregulation of coproporphyrinogen III oxidase (CPOX) by differentiation agents such as vitamin D [24, 25] and genetic modulation of FECH [20, 26] and ABCB6 [23] all led to enhanced ALA-induced PpIX production in tumor cells, underlining the importance of these molecules in promoting PpIX biosynthesis and accumulation in tumors. There are studies showing that cell transformation by transfecting normal cells with oncogenes such as Ras and polyomavirus middle T antigen increased ALA-induced PpIX fluorescence [27, 28]. Although how oncogene transformation elevated PpIX level was not reported, results of these studies suggest an association between oncogene transformation and enhanced ALA-induced PpIX production. As oncogenesis is a cell transformation process activated by various oncogenes, identifying a causal relationship between oncogene transformation and enhanced PpIX biosynthesis, and understanding the mechanism involved are important for selecting appropriate patients for ALA-based modalities.

We examined the effects of human epidermal growth factor receptor (Her2, ERBB2 or Neu in rats) oncogene transformation on ALA-induced PpIX fluorescence and PDT response in this study. Her2/NeuT, encoded by *ERBB2* gene, is a transmembrane tyrosine kinase receptor expressed on a variety of cells [29]. It belongs to ERBB protein family that includes four members (Her1-4 or ERBB1-4), all of which are receptor tyrosine kinases. As a driver oncogene in cancer formation, Her2/Neu aberrations, particularly through gene amplification, are involved in a variety of human cancers including breast, gastric, pancreatic, ovarian and non-small cell lung cancers [30]. About 20% breast cancer patients exhibit Her2/Neu overexpression due to gene amplification [31]. To the best of our knowledge, the effect of Her2/Neu oncogene transformation on ALA-induced PpIX and PDT response has never been studied. Here we report that Her2/Neu oncogene transformation enhanced ALA-induced PpIX fluorescence and altered PpIX intracellular localization. As a result, Her2/Neu-transformed cells showed increased sensitivity to ALA-mediated PDT. Our results provide a foundation for using ALA as a dual imaging and PDT agent for Her2/Neu-transformed tumors.

## RESULTS

### NeuT oncogene expression transformed MCF10A human breast epithelial cells

**Figure 1:**
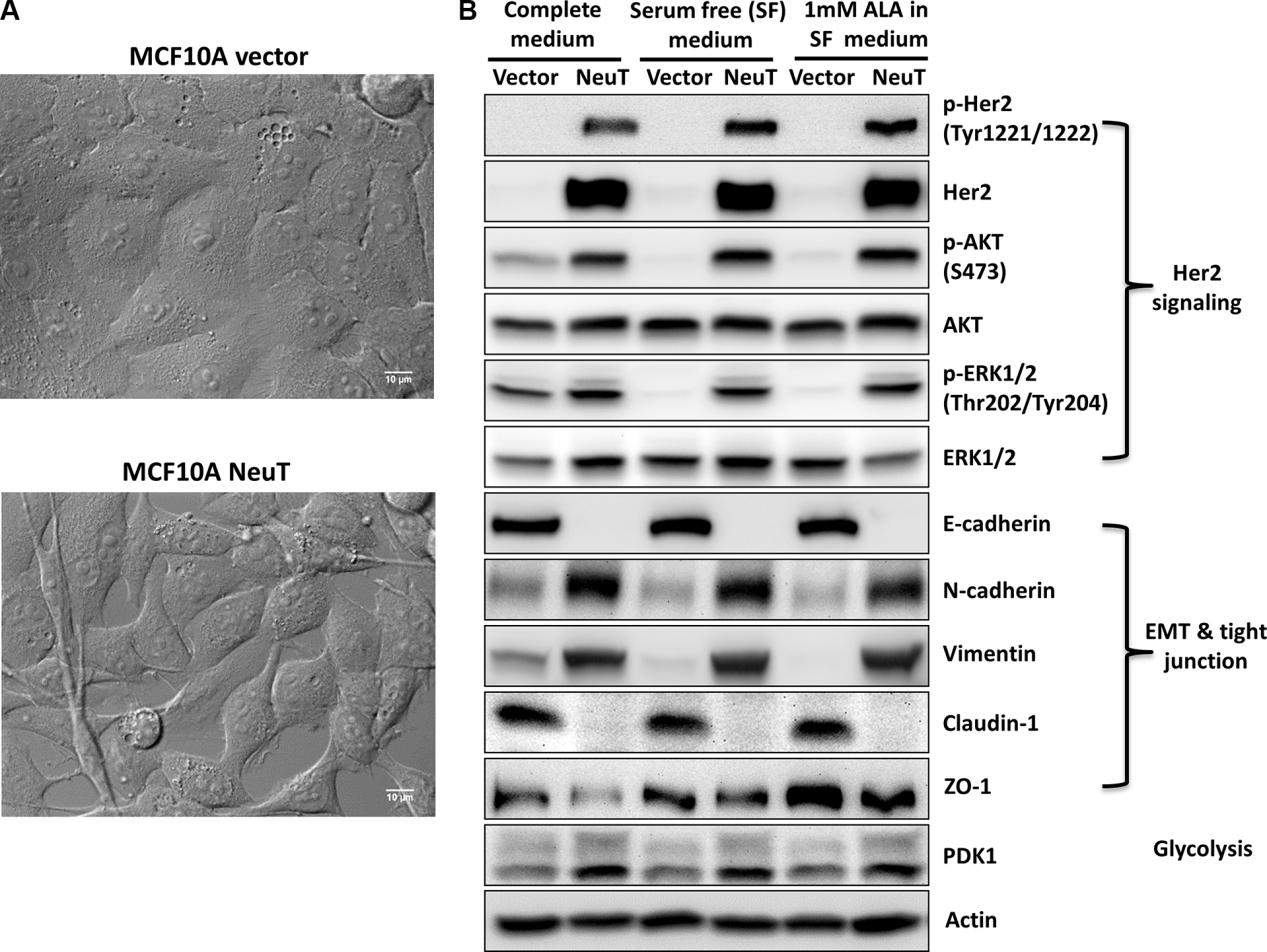
Her2/NeuT oncogene expression transformed MCF10A human breast epithelial cells (**A**) Differential interference contrast (DIC) images (60×) show distinct differences in cell morphology between MCF10A vector control and NeuT-transformed cells. (**B**) Her2/NeuT oncogene transformation altered cell signaling. MCF10A vector and NeuT cells were cultured in complete DMEM/F12 medium, serum free medium with or without 1 mM ALA for 4 h and lysed with lysis buffer. Cell lysates were examined by Western blot for Her2/Neu signaling molecules, EMT and tight junction markers, and glycolytic enzyme PDK1.

Expression of NeuT, a mutated Her2/Neu with enhanced tyrosine kinase activity [32], in MCF10A human breast epithelial cells caused significant changes in cell morphology. As shown in Figure 1A, MCF10A vector cells exhibit well organized cobblestone epithelial cell shape whereas NeuT-transformed cells show poorly organized, elongated and motile fibroblast cell morphology. In agreement with morphological changes, significant alterations in cell signaling were found in NeuT-transformed cells compared with vector control cells (Figure 1B). Expression of NeuT induced receptor autophosphorylation, which activated AKT and ERK signaling, two major Her2/Neu downstream signaling pathways involved in cell proliferation and migration. NeuT oncogene induced epithelial-mesenchymal transition (EMT) as indicated by the loss of epithelial marker E-cadherin and increased level of mesenchymal markers N-cadherin and vimentin in MCF10A NeuT cells. NeuT cells also lost the expression of tight junction molecule claudin-1 and had reduced level of another tight junction molecule ZO-1 compared with vector cells. Furthermore, NeuT transformation induced the up-regulation of pyruvate dehydrogenase kinase 1 (PDK1), an important enzyme involved in the inhibition of glucose oxidation in mitochondria and the switch to glycolytic metabolism [33].

### NeuT oncogene transformation enhanced ALA-induced PpIX fluorescence

**Figure 2:**
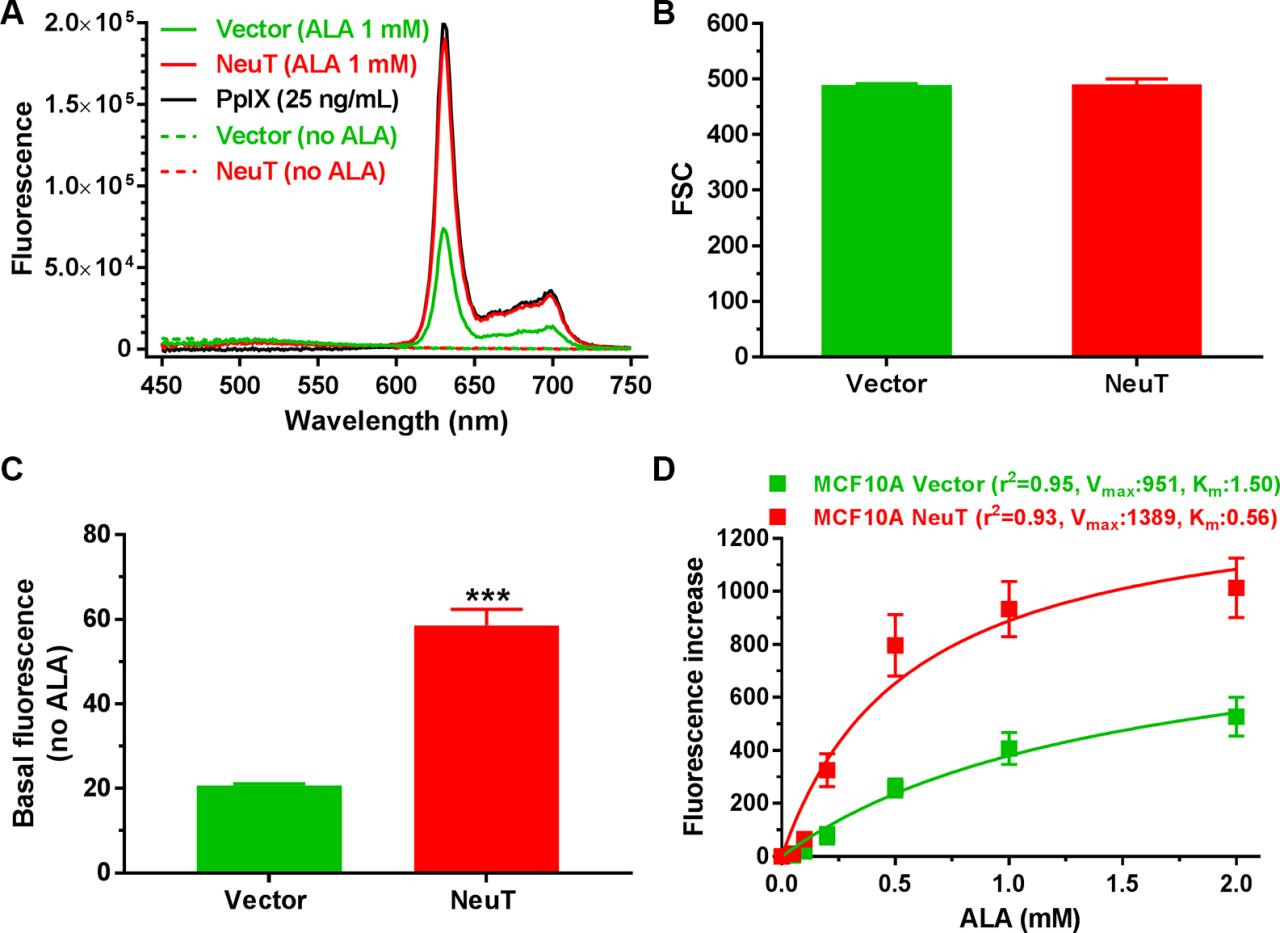
NeuT oncogene transformation enhanced ALA-induced PpIX fluorescence (**A**) Fluorescence spectra of MCF10A vector and NeuT cell lysates and PpIX standard (25 ng/mL in DMSO). Both vector and NeuT cells were incubated with 1 mM ALA in serum free medium for 4 h and lysed. Fluorescence spectra of cell lysates and PpIX standard were obtained using 400 ± 2.5 nm excitation. (**B**–**D**) Flow cytometer analysis showing forward scatter (FSC) (B), basal cell fluorescence without ALA (C), and a dose-dependent fluorescence increase after ALA incubation (D) in MCF10A vector and NeuT cells. Cells were cultured in serum free DMEM/F12 medium with or without ALA for 4 h and cell fluorescence was measured with flow cytometry. ALA-induced fluorescence increase, calculated by subtracting basal cell fluorescence without ALA from cell fluorescence after incubation with different doses of ALA, was fit with the Michaelis-Menten enzyme kinetics. Data are presented as mean ± SD from at least 3 experiments. ***p < 0.001 compared with vector cells.

Fluorescence spectra of MCF10A vector and NeuT cell lysates after 4 h incubation with 1 mM ALA in serum free medium were shown in Figure 2A. The fluorescence spectrum of NeuT cell lysate overlapped with that of PpIX standard, suggesting that PpIX was the predominant porphyrin metabolite accumulated in NeuT cells following ALA incubation. ALA also caused PpIX accumulation in vector cells because similar fluorescence spectrum was detected in the vector cell lysate. But ALA-induced PpIX fluorescence in NeuT cell lysate was much higher than in the vector cell lysate. PpIX fluorescence emission peaks were not detectable in MCF10A vector and NeuT cell lysates without ALA treatment.

To compare fluorescence intensity between vector and NeuT cells, cells were incubated without or with ALA for 4 h in serum free medium and cell fluorescence was quantified by a flow cytometer in the FL3 channel (488 nm excitation, 650 nm long pass emission). Analysis of flow cytometer forward scatter parameter, an indicator of cell size, showed no significant difference between vector and NeuT cells (*p* > 0.05, Figure 2B). NeuT cells had a significantly higher basal fluorescence (without ALA) than vector cells (*p* < 0.001, Figure 2C). ALA incubation caused a dose-dependent fluorescence increase in both vector and NeuT cells, but fluorescence increase in NeuT cells was much greater than in vector cells (Figure 2D). Interestingly, ALA-induced fluorescence increase in both cell lines fit well into the Michaelis-Menten enzyme kinetics. Compared with vector cells, NeuT cells showed a higher V_max_ (the maximum ALA-induced PpIX fluorescence after 4 h incubation) and lower K_m_ (the ALA concentration at the half-maximum PpIX fluorescence), suggesting that NeuT oncogene transformation increased the capacity of ALA-induced PpIX production.

### NeuT oncogene transformation elevated the level of heme biosynthesis enzymes

**Figure 3:**
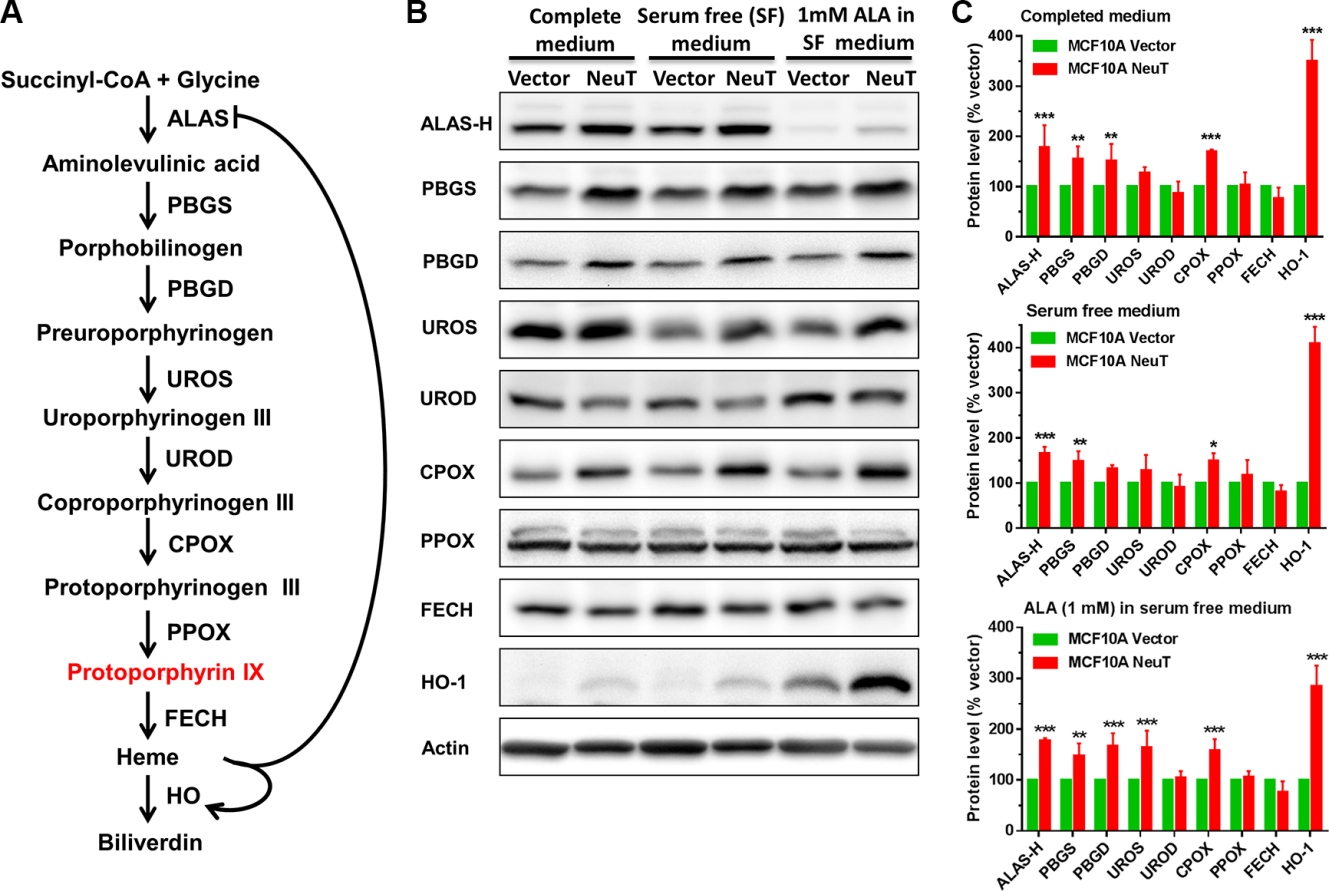
NeuT oncogene transformation elevated the level of heme biosynthesis enzymes (**A**) Heme biosynthesis and degradation pathway. Heme biosynthesis pathway is composed of eight enzymatic steps, which begins with the synthesis of ALA by ALAS and ends with the conversion of PpIX to heme by FECH. Heme is degraded by HO. Heme homeostasis is achieved by balancing its biosynthesis and degradation, which is regulated by heme level. (**B** and **C**) Effects of NeuT oncogene transformation on heme biosynthesis and degradation enzymes. (B) MCF10A vector and NeuT cells in complete medium, serum free medium with or without ALA (1 mM for 4 h) were lysed and probed for heme biosynthesis and degradation enzymes by Western blot. (C) Quantification of Western blot band intensity showing enzyme level changes in NeuT cells relative to vector cells (normalized to 100%). Data are presented as mean ± SD from at least 3 experiments. * *p* < 0.05, **p < 0.01, ***p < 0.001 compared with vector cells.

To determine the cause of increased PpIX level in NeuT cells, heme biosynthesis and degradation enzymes were examined by Western blot analysis and compared between vector and NeuT cells (Figure 3). As shown in Figure 3A, heme biosynthesis pathway is composed of 8 enzymes including ALA synthase (ALAS), porphobilinogen synthase (PBGS), porphobilinogen deaminase (PBGD), uroporphyrinogen III synthase (UROS), uroporphyrinogen III decarboxylase (UROD), coproporphyrinogen III oxidase (CPOX), protoporphyrinogen III oxidase (PPOX) and ferrochelatase (FECH). Heme is synthesized in mitochondria from PpIX and ferrous iron by FECH and degraded into biliverdin by heme oxygenase (HO) [34]. Heme biosynthesis and degradation is tightly regulated by the heme level, which inhibits heme biosynthesis by imposing negative feedback inhibition on the rate-limiting enzyme ALAS and stimulates heme degradation by promoting the expression of heme degradation enzyme HO [34].

Compared with vector cells, NeuT transformation significantly increased the protein level of some enzymes (Figure 3B, 3C). Particularly, levels of ALAS-H, PBGS, CPOX and HO-1 were significantly increased in all three culture conditions. HO-1 showed the highest relative change induced by NeuT transformation. PBGD level was significantly increased when cells were cultured in completed medium and serum free medium with ALA. UROS level was significantly increased only when cells were cultured with ALA in serum free medium. Three enzymes UROD, PPOX and FECH showed no significant change between vector and NeuT cells. It is important to note that ALA treatment down-regulated ALAS-H and up-regulated HO-1, indicating a feedback regulation of heme biosynthesis and degradation pathway by pathway product heme.

### ALA-induced PpIX exhibited mitochondrial localization at a low cell density and cell membrane localization at a high cell density in vector cells whereas it showed a strong mitochondrial localization in NeuT cells regardless of cell density

**Figure 4:**
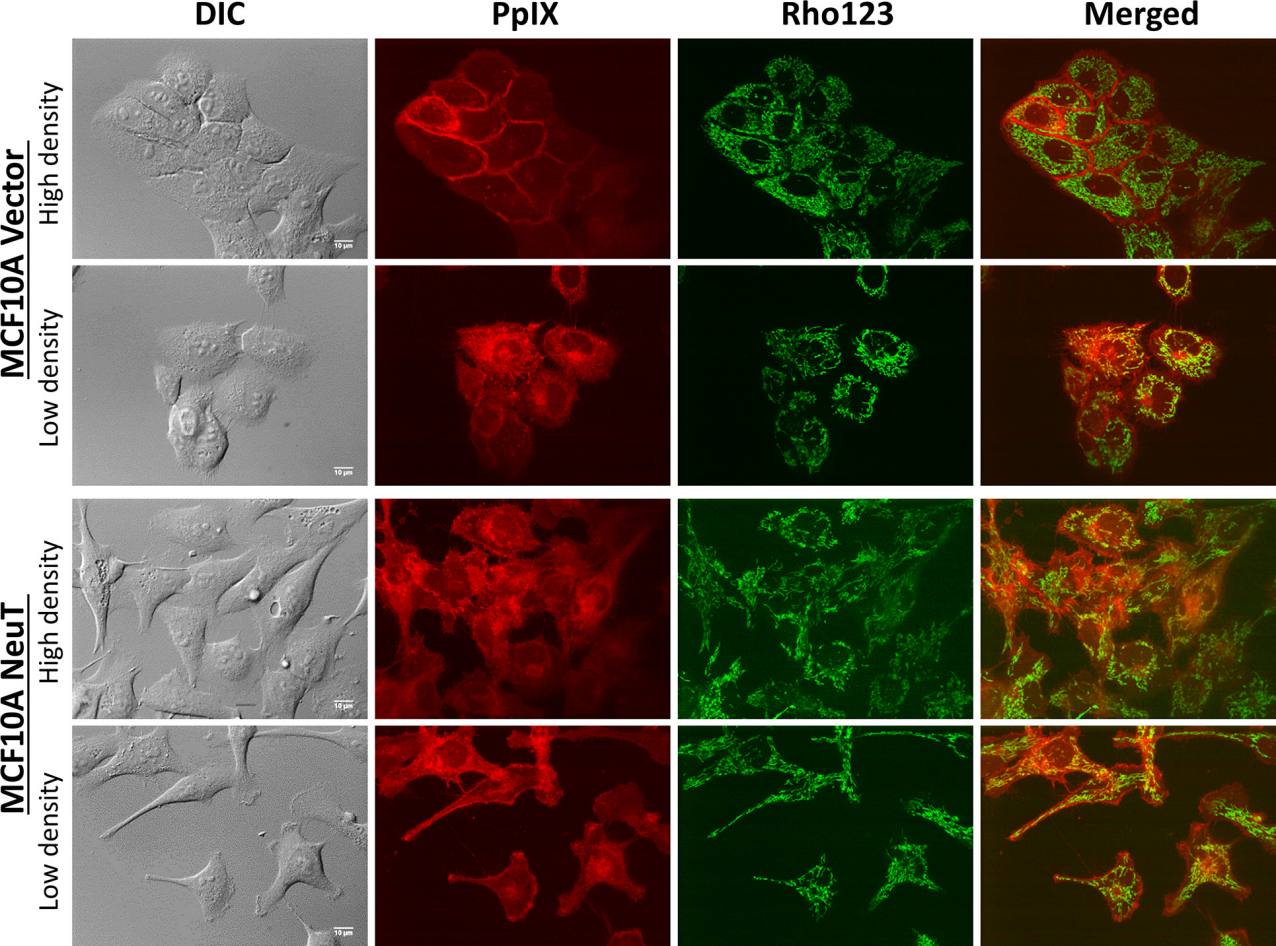
NeuT oncogene transformation altered ALA-induced PpIX intracellular localization from being cell contact-dependent membrane localization to being mitochondrial localization independent of cell density MCF10A vector and NeuT cells were incubated with 1 mM ALA in serum free medium for 4 h and imaged with a confocal fluorescence microscope for ALA-induced PpIX and mitochondrial marker rhodamine 123 (250 ng/mL for 30 min). Images of vector and NeuT cells at both high and low cell densities/contact are shown. Bars, 10 μm.

**Figure 5:**
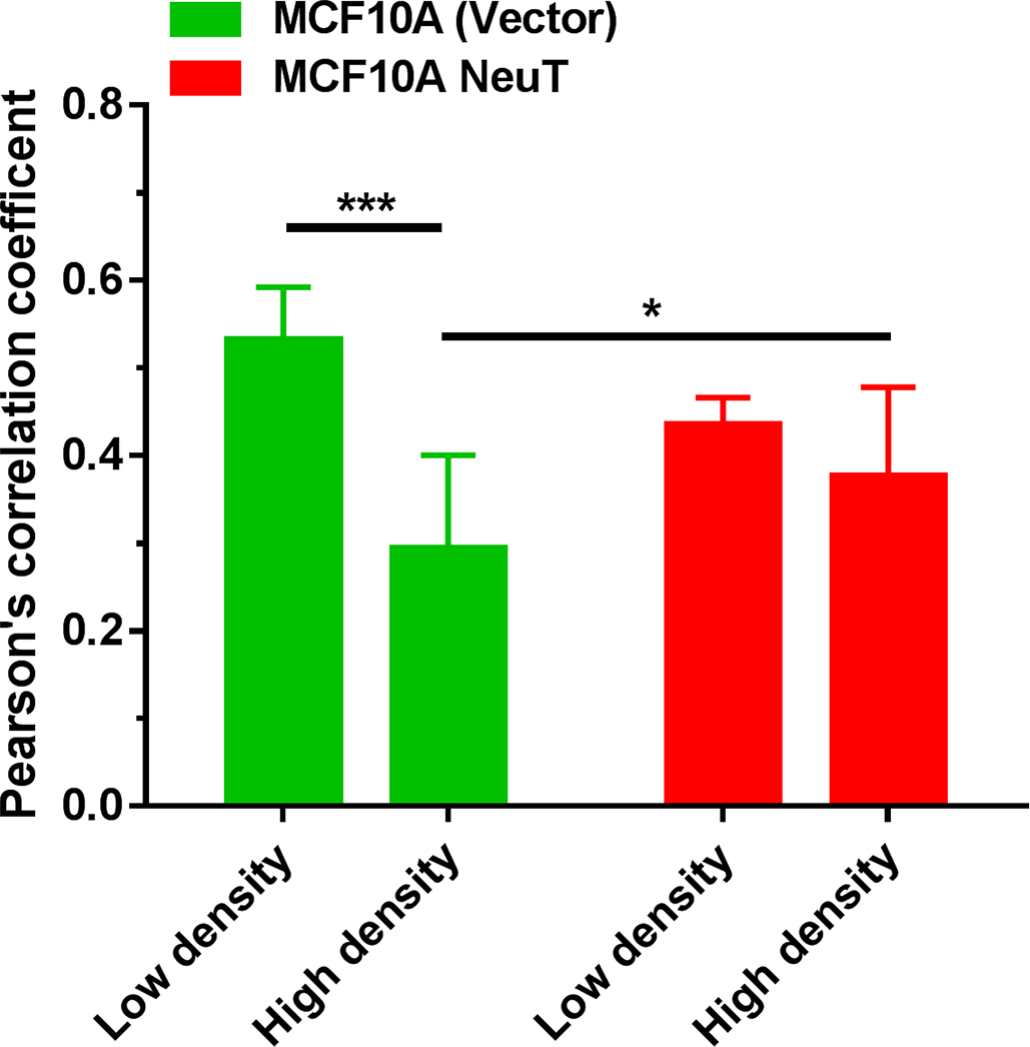
Co-localization between ALA-induced PpIX and mitochondrial marker rhodamine 123 fluorescence in MCF10A (vector) control and NeuT-transformed cells MCF10A (vector) and NeuT cells were imaged with a confocal microscope for ALA-induced PpIX and rhodamine 123 as described in Figure 4. Pearson's correlation coefficients between PpIX and rhodamine 123 fluorescence images were analyzed. Images with less 10 cells are pooled in the low density group and images with more than 10 cells are pooled in the high density group. At least 7 images were included in each group. *p < 0.05, ***p < 0.001.

**Figure 6:**
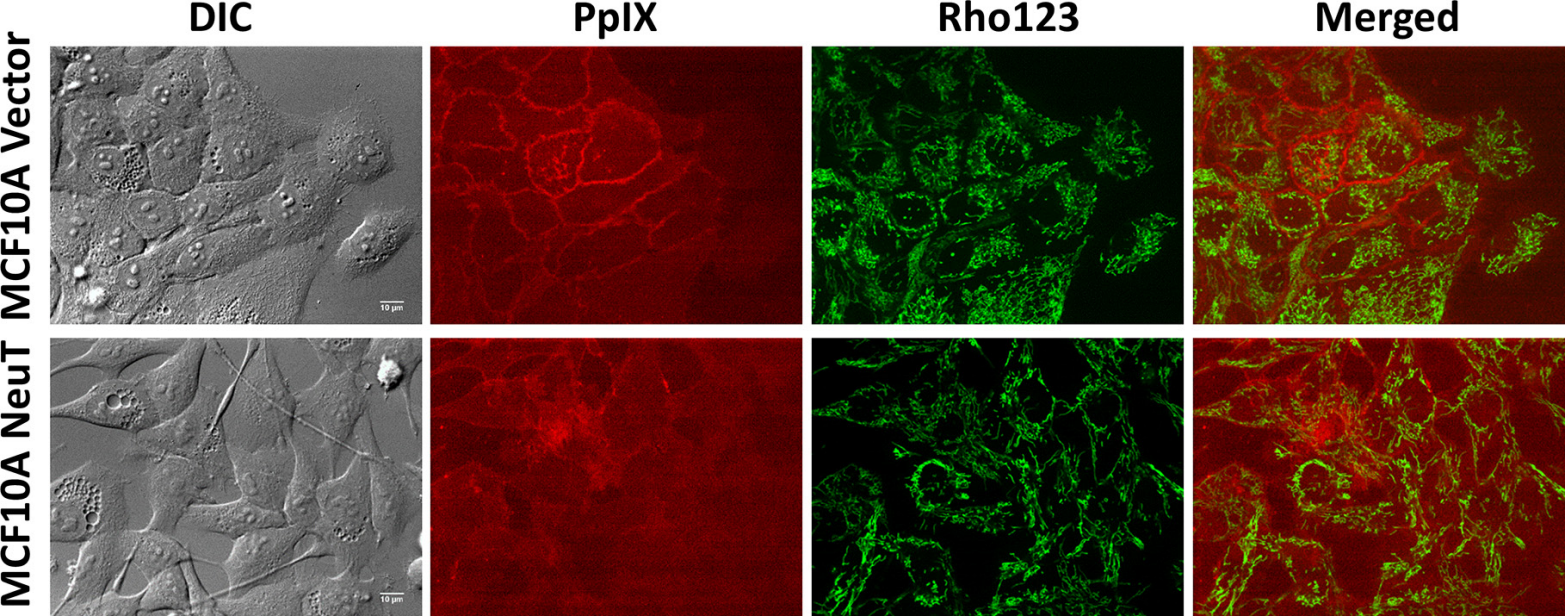
Exogenous PpIX showed cell contact-dependent membrane localization in MCF10 A vector cells, but not in the NeuT cells MCF10A vector and NeuT cells were incubated with 100 ng/mL PpIX in serum free medium for 4 h and imaged with a confocal fluorescence microscope. Mitochondria were labelled by incubating cells with rhodamine 123 (250 ng/mL) for 30 min. Bars, 10 μm.

The intracellular localization of PpIX in relation to mitochondria highlighted by mitochondrial marker rhodamine 123 was examined by confocal microscopy in MCF10A vector and NeuT cells after 4 h incubation with 1 mM ALA in serum free medium (Figure 4). Co-localization between PpIX and rhodamine 123 fluorescence images, as indicated by the Pearson's correlation coefficient between 2 channels, was analyzed by NIH ImageJ software and shown in Figure 5. In vector cells at a high cell density, PpIX fluorescence was seen primarily associated with the cell membrane where cells maintained contact with neighboring cells and much less PpIX fluorescence was observed at membrane area without cell contact (Figure 4). But at a low cell density with a little cell contact, PpIX fluorescence was found localized in mitochondria. Co-localization analysis showed that PpIX fluorescence in vectors cells at low cell densities exhibited a significantly higher co-localization with mitochondria than cells at high cell densities (Figure 5). In contrast to vector cells, cell contact/density did not appear to affect ALA-induced PpIX intracellular localization in NeuT cells because cells at both low and high cell densities exhibited similar PpIX mitochondrial localization (Figures 4, 5). In addition, the co-localization between PpIX and mitochondria was significantly higher in NeuT cells than in vector cells at high cell densities, but showed no significant difference at low cell densities (Figure 6).

We also incubated vector and NeuT cells with exogenous PpIX (100 ng/mL) for 4 h in serum free medium and compared the localization of exogenously applied PpIX with endogenously produced PpIX from ALA. Similar to the localization of ALA-induced PpIX in vector cells (Figure 4), the fluorescence of exogenous PpIX was mainly observed on vector cell membrane with neighboring cell contact, and much reduced fluorescence was seen on cell membrane without cell contact (Figure 6). This cell contact-dependent membrane localization of PpIX was absent in NeuT cells treated with exogenous PpIX, as in NeuT cells treated with ALA.

### NeuT-transformed cells were more sensitive to PDT with ALA than vector cells

**Figure 7:**
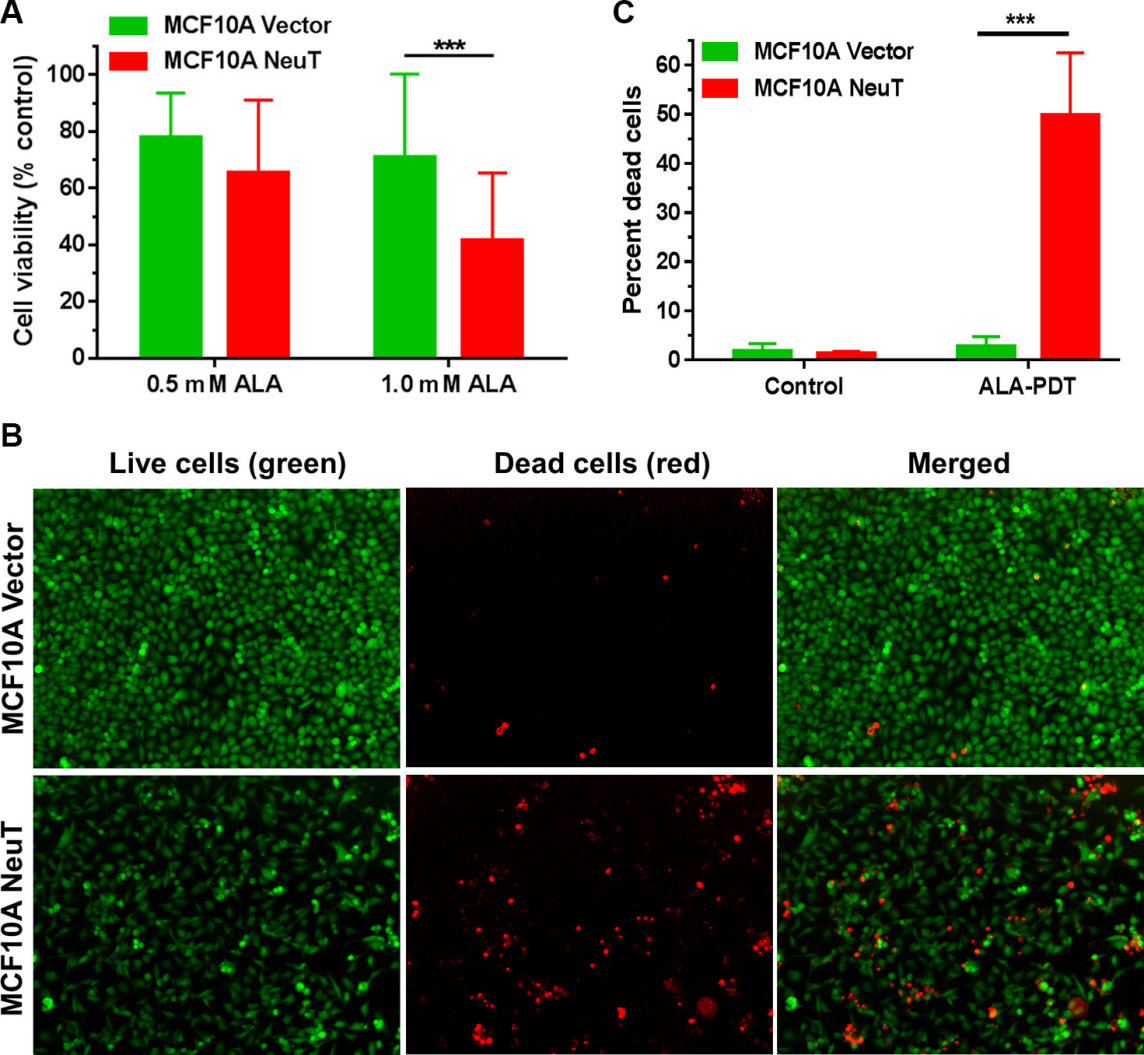
NeuT oncogene transformation increased cell sensitivity to ALA-mediated PDT (**A**) Effects of ALA-mediated PDT on cell viability. MCF10A vector and NeuT cells were treated with 3 J/cm^2^ light of 633 nm after 4 h incubated with 0.5 or 1.0 mM ALA in serum free medium. Cell viability was measured with the MTS assay at 24 h after treatment (*n* = 7). *** *p* < 0.001. (**B** and **C**) Live/Dead cell viability assay showing cell death after ALA-mediated PDT. MCF10A vector and NeuT cells were labelled with Live/Dead cell viability/cytotoxicity kit at 24 h after PDT (1 mM ALA for 4 h, 3 J/cm^2^). (B) Fluorescence images showing live and dead cells after PDT. (C) Image quantification of percent dead cells after treatments. Experiments were repeated 3 times. One representative experimental results are shown. Each treatment condition includes 4–6 images. ***p < 0.001.

Effects of PDT with ALA on MCF10A vector and NeuT cell viability were examined by the MTS assay at 24 h after treatment. Figure 7A shows that PDT (3 J/cm^2^) with 1 mM ALA causes significantly more reduction in cell viability in NeuT cells than in vector cells (*p* < 0.001). PDT with 0.5 mM ALA also induced more cell viability decrease in NeuT cells than in vector cells, although the difference was not statistically significant (*p* > 0.05). To visualize live and dead cells after treatments, cells were labelled with Live/Dead cell viability/cytotoxicity kit at 24 h after PDT (3 J/cm^2^) with 1 mM ALA and imaged with a fluorescence microscope. Compared with vector control cells, more dead cells (red) were found in NeuT cells after PDT (Figure 7B). Image quantification of dead cells over the total number of cells in each fluorescence microscopic image indicate that PDT caused significantly more dead cells in NeuT cells than in vector cells (*p* < 0.001, Figure 7C).

## DISCUSSION

Effects of Her2/Neu oncogene, a driver oncogene in human cancers, on ALA-induced PpIX fluorescence, PpIX intracellular localization and cell sensitivity to ALA-mediated PDT were studied in the present study. Our results demonstrate that transfecting MCF10A human breast epithelial cells with NeuT oncogene caused cell transformation by activating Her2/Neu signaling, inducing EMT, and altering glucose metabolism. NeuT-transformed cells not only exhibited a higher ALA-induced PpIX fluorescence than vector control cells, but also displayed a stronger mitochondrial localization of PpIX. Consequently, NeuT cells showed increased response to PDT with ALA than vector cells. These results reveal a link between NeuT oncogene transformation and enhanced ALA-induced PpIX production, and strongly support the use of ALA for imaging and targeting Her2-positive tumors.

NeuT transformation significantly increased the basal fluorescence (without ALA) as well as ALA-induced PpIX fluorescence in MCF10A cells (Figure 2). Because PpIX fluorescence peaks were not detectable in the basal fluorescence emission of cell lysates, we were unable to conclude that NeuT transformation increased PpIX level without ALA stimulation. Analytical tools with higher sensitivity will be needed to determine whether increased basal fluorescence in NeuT cells is indeed due to PpIX. Nevertheless, spectrofluorometric analysis did demonstrate that PpIX was the predominant porphyrin metabolite accumulated in both NeuT and vector control cells after ALA stimulation based on the detection of characteristic PpIX fluorescence emission spectrum. Fluorescence measurements by spectrofluorometer and flow cytometer showed that PpIX fluorescence in NeuT cells was more than two times as much as in vector cells after ALA (1 mM) treatment.

To determine the possible cause of increased ALA-induced PpIX in NeuT versus vector cells, we compared the cell size between the two cell lines because we previously reported that ALA-induced PpIX fluorescence in different cell lines was related to the cell size [35], and EMT is known to cause cell size changes [36]. Using flow cytometer forward scatter parameter as the indicator of cell size, we found that NeuT and vector control cells had almost identical forward scatter. Since ALA-induced PpIX is dependent on heme biosynthesis and degradation pathway, the level of enzymes involved in heme biosynthesis and degradation was compared between NeuT and vector cells by Western blot analysis. Here we found that NeuT oncogene transformation induced a significant up-regulation of some heme biosynthesis enzymes including ALAS-H, PBGS, PBGD and CPOX (Figure 3). Increased PBGS, PBGD and CPOX, but not ALAS-H, in NeuT cells would enhance ALA-induced PpIX/heme production because exogenous ALA bypassed the action of ALAS-H and its enzymatic activity was inhibited in the presence of exogenous ALA due to heme-mediated negative feedback inhibition. Since PpIX bioconversion enzyme FECH level was not significantly changed, we think that increased PpIX biosynthesis due to the upregulation of PpIX biosynthesis enzymes outpaced its conversion to heme, resulting in greater PpIX accumulation in NeuT cells.

Previous studies have shown that increased PBGD activity in cancer over normal cells [21, 37, 38] and after ALA stimulation [18, 19] was associated with increased ALA-induced PpIX production. Increased CPOX level induced by differentiation agents such as methotrexate [39, 40] enhanced cell PpIX production after ALA treatment. Moreover, reduced FECH expression and activity in tumors were shown to cause PpIX accumulation by diminishing PpIX conversion to heme [20–22]. Our present study suggests that NeuT oncogene largely increased ALA-induced PpIX through enhanced PpIX production rather than reduced PpIX conversion. The finding that multiple PpIX biosynthesis enzymes such as PBGS, PBGD and CPOX were upregulated after NeuT transformation indicates a profound heme pathway upregulation. It is known that NeuT oncogene activates a variety of transcription factors including activator protein 1 (AP-1) and NF-κB [41, 42], which are shown to regulate the transcription of heme biosynthesis enzymes such as CPOX [25]. Thus, the upregulation of PpIX biosynthesis enzymes is likely a consequence of NeuT oncogene-mediated transcriptional activation.

In addition to enhancing ALA-induced PpIX fluorescence, NeuT oncogene transformation was found to alter the intracellular localization of PpIX (Figures 4, 6). Both endogenous and exogenous PpIX exhibited cell contact-dependent membrane localization in vector cells but not in NeuT cells, which may suggest that PpIX was associated with certain cell surface molecules that are involved in cell contact/adhesion and do not express in NeuT-transformed cells. Although the identity of these PpIX-bound cell surface molecules remains to be determined, they can be epithelial adhesion molecules such as E-cadherin or tight junction molecules such as claudin-1 as these molecules were found only expressed in vector cells (Figure 1). The notion that PpIX is associated with cell surface molecules involved in cell contact/adhesion is supported by previous reports that PDT with ALA inhibited cell attachment even at a non-lethal dose [43, 44]. It is possible that cell attachment inhibition is caused by PDT-induced direct photo-damage to cell contact/adhesion molecules due to the association between PpIX and these cell surface molecules.

Confocal microscopic imaging also revealed that, different from cell contact-dependent membrane localization of PpIX in vectors cells, NeuT cells showed a consistent mitochondrial localization of ALA-induced PpIX regardless of cell density. PpIX is commonly detected in mitochondria in tumor cells after ALA and, because of this preferential localization, the mitochondrion has been considered as a sensitive target of ALA-mediated PDT [45, 46]. However, the cause of PpIX mitochondrial localization in tumor cells remains poorly defined. Since PpIX and other porphyrin metabolites are cytotoxic due to their photosensitive and oxidative activities, cells normally maintain free porphyrins at a low level by pumping excess porphyrins out of cells with transporters localized on mitochondrial and cell membranes [47]. More PpIX mitochondrial accumulation in NeuT cells than in vector cells suggests NeuT oncogene-induced alterations in mitochondrial function and porphyrin transporter activity. As we found in the present study, NeuT transformation increased the expression of PDK1, which inhibits the conversion of pyruvate to acetyl-CoA for tricarboxylic acid (TCA) cycle [33]. Previous studies have shown that Her2/Neu overexpression prompts pyruvate to lactate conversion by upregulating the expression of lactate dehydrogenase A (LDH-A) [48], and NeuT oncogene activates glutaminolysis by increasing glutaminase 1 expression [49]. All these results indicate that Her2/Neu transformation suppresses mitochondrial oxidative phosphorylation and switches energetic metabolism to aerobic glycolysis. As recent evidence suggests an association between tumor glycolysis and enhanced ALA-induced PpIX accumulation [50], it is possible that NeuT-induced tumor glycolysis contributes to enhanced mitochondrial localization of PpIX.

An important finding of our study is that NeuT-transformed cells showed increased sensitivity to ALA-mediated PDT than vector cells (Figure 7), likely due to increased PpIX production and mitochondrial localization. In agreement with this result, we have shown previously that two Her2-postitive breast cancer cell lines SkBr3 and MDA-MB-453 are more responsive to PDT with ALA than MCF10A breast epithelial cells [11]. Because Her2/Neu overexpression not only drives breast cancers but also activates the oncogenesis of a variety of other types of human cancers, future studies should determine whether ALA-based modalities are effective for non-breast Her2-overexpressed tumors. It is also necessary to evaluate whether tumor cell response to ALA-mediated PDT can be predicted by Her2 expression. As Her2/Neu overexpression as well as EMT are often associated with cancer chemoresistance [51, 52], our present finding that NeuT-transformed cells were sensitive to ALA-mediated PDT together with previous studies of PDT on chemoresistant cancer cells (as reviewed in [53]) highlights the promise of using PDT to overcome cancer chemoresistance.

In summary, results presented in this study demonstrate that NeuT oncogene transformation enhanced ALA-induced PpIX production and promoted PpIX mitochondrial localization. As a result, NeuT-transformed cells became more sensitive to ALA-mediated PDT than vector control cells. Our study demonstrates that Her2/Neu oncogene represents a target for ALA-based imaging and therapeutic modalities.

## MATERIALS AND METHODS

### Chemicals and reagents

Delta-aminolevulinic acid hydrochloride (ALA), purchased from Frontier Scientific Inc. (Logan, UT), was dissolved in phosphate buffered saline (PBS) solution. Protoporphyrin IX (PpIX) from Frontier Scientific Inc. was dissolved in DMSO solution. The solution of chemicals was sterilized by passing through filters and stored in a −20^°^C freezer. They were directly added into cell culture medium for treatment. The final concentration of DMSO in the medium was 0.1% or less. Live/Dead cell viability/cytotoxicity kit was purchased from Thermo Fisher Scientific and used according to manufacturer's instruction.

### Cell culture and transfection

MCF10A human breast epithelial cells were routinely maintained in complete DMEM/F12 (50/50) medium (Mediatech, Manassas, VA) supplemented with 5% horse serum (Atlanta Biologicals), insulin 10 ug/mL, epidermal growth factor (EGF) 20 ng/mL, cholera toxin 100 ng/mL, hydrocortisone 0.5 ug/mL and 1% of antibiotics and antimycotics solution (Mediatech) at 37^°^C in a humidified 5% CO_2_ incubator. Serum free (SF) DMEM/F12 medium was basal medium with supplement of antibiotics and antimycotics only.

The method for establishing MCF10A vector and MCF10A NeuT cells was described previously [54]. Briefly, retroviral vector plasmids encoding NeuT (a mutated Her2/Neu with enhanced tyrosine kinase activity) were introduced into HEK 293T cells by transient co-transfection with helper viral vector and calcium phosphate precipitation. After 6 h, cell culture medium was replaced with fresh medium and cells were allowed to grow for 36 h to produce viruses. The supernatant was then collected and filtered through a 0.45-um filter. MCF10A cells were infected at approximately 70% confluence in serum free DMEM/F12 medium supplemented with 8 ug/ml of polybrene. On the following day, medium was changed to complete DMEM/F12 medium. To select stably transfected cells, cells were incubated in complete DMEM/F12 medium supplemented with 2 μg/ml puromycin for two weeks followed by continuous passaging for at least 20 passages. The protein level of NeuT was monitored by Western blot as described below. Stable MCF10A vector and NeuT cells were maintained in complete DMEM/F12 medium.

### Spectrofluorometry

MCF10A vector and NeuT cells were implanted in cell culture dishes and allowed to grow for 2 days. Cells were then incubated with serum free DMEM/F12 medium with or without 1 mM ALA for 4 h. After the medium was removed, cells were rinsed with PBS, trypsinized and counted. Equal number of vector and NeuT cells (4 × 10^5^) were taken and pelleted. Cell pellets were suspended in DMSO and sonicated for 30 min. Cell lysates were centrifuged to obtain supernatants for spectrofluorometric analysis. Fluorescence emission spectra of cell lysates as well as PpIX standard were measured with a Fluoromax-3 fluorescence spectrometer (Horiba JY, Edision, NJ) using 400 ± 2.5 nm excitation.

### Flow cytometry

Cellular PpIX fluorescence intensity was determined by flow cytometry. MCF10A vector and NeuT cells were seeded in cell culture dishes and grown in complete DMEM/F12 medium for two days to reach about 70% confluence. After removing the medium, cells were rinsed with PBS twice and then incubated with serum free DMEM/F12 medium containing ALA for ALA-treated cells or no ALA for control cells. At the end of 4 h incubation, cells were rinsed with PBS, trypsinized, and suspended in PBS. Cell suspensions were centrifuged and cell pellets were re-suspended in PBS for flow cytometer measurement. Cellular fluorescence in the FL3 channel (488 nm excitation, 650 nm long pass emission) was measured with a FACSCalibur flow cytometer (BD Biosciences). About 20,000 cells were measured and recorded for each experiment. Experiments were repeated at least three times.

### Western blot

Western blot was performed as described previously [55]. Briefly, MCF10A vector and NeuT cells were treated and lysed in NP40 lysis buffer supplemented with protease and phosphatase inhibitors. Cell lysates were separated by sodium dodecyl sulphate polyacrylamide gel electrophoresis (SDS-PAGE) and then electrophoretically transferred to polyvinylidene fluoride (PVDF) membranes. Blots were incubated with primary antibodies and followed by incubation with horseradish peroxidase-conjugated secondary antibody from Cell Signaling (Danvers, MA). Primarily antibodies against ALAS-H, PBGS, UROD and PPOX were purchased from Santa Cruz (Dallas, TX). HMBS, UROS, CPOX and FECH primarily antibodies were obtained from Abcam (Cambridge, MA). Immunoblots were incubated with SuperSignal West Dura extended duration substrate (Thermo Scientific) and immunoreactive bands were captured with an UVP imaging system (UVP LLC, Upland, CA). The density of immunoreactive bands was qualified with NIH ImageJ software and normalized to the corresponding actin band intensity for analysis.

### Confocal fluorescence microscopy

PpIX fluorescence was imaged with confocal fluorescence microscopy as described previously [56]. MCF10A vector and NeuT cells were grown in complete DMEM/F12 medium in glass bottom cell culture dishes (MatTek). Cells were rinsed with PBS twice and incubated with serum free DMEM/F12 medium containing ALA (1 mM) or PpIX (100 ng/mL) for 4 h. Mitochondrial marker rhodamine 123 (Thermo Fisher) was added into the medium at 30 min before due time to label mitochondria. After the incubation, cells were washed with PBS three times and incubated in serum free medium for confocal imaging.

Live-cell imaging was performed on a Nikon TiE (Eclipse) confocal microscope using a 60× oil immersion objective equipped with a CSU-X spinning disk confocal scan head (Yokogawa), a temperature-controlled linear encoded robotic stage (ASI Technologies, Inc.), a multi-bandpass dichromatic mirror (Semrock) and bandpass filters (Chroma Technology Corp.) in an electronic filter wheel. Microscope system was appropriately set to image the fluorescence of PpIX (405 nm excitation, 700 ± 37.5 nm emission) and rhodamine 123 (488 nm excitation, 525 ± 18 nm emission). Laser illumination was provided by a 50 mW monolithic laser combiner (MLC400, Agilent Technologies) and images were acquired using a Clara interline CCD camera (Andor Technology). Depending on the fluorophore, exposure time ranged from 200-500 ms. Differential interference contrast (DIC) images were acquired using exposure times in the range of 100–200 ms at the same magnification. PpIX and rhodamine 123 fluorescence images were pseudo-colored and merged to generate composite images. Pearson's correlation coefficient between PpIX and rhodamine 123 fluorescence images were analyzed to indicate the extent of co-localization. Pearson's correlation coefficient ranges from 0 (no co-localization) to 1 (100% co-localization). All image processing and analysis were performed with NIH ImageJ software.

### PDT treatment and cytotoxicity assay

MCF10A vector and NeuT cells were implanted in 96-well plates in complete DMEM/F12 medium. After cells were rinsed with serum free DMEM/F12 medium, they were incubated in serum free medium containing ALA (0.5 or 1.0 mM) or no ALA (for control) for 4 h. Cells were then treated with 5 mW/cm^2^ irradiance of 633 nm light for 10 min, which resulted in a light fluence of 3.0 J/cm^2^. Light illumination was provided by a diode laser system (High Power Devices Inc., North Brunswick, NJ) coupled to a 600 μm core diameter optical fiber fitted with a microlens at the end of fiber to achieve homogeneous irradiation. Light intensity was measured with an optical power meter (Thorlabs, Inc., North Newton, NJ). Immediately after light treatment, serum free medium was replaced with complete DMEM/F12 medium for culture. Cell viability was determined at 24 h after treatment using CellTiter 96 Aqueous Non-Radioactive Cell Proliferation Assay (MTS assay, Promega, Madison, WI). Effects of PDT on cell survival and death were also assessed with a fluorescence microscope after incubating cells with Live/Dead cell viability/cytotoxicity kit reagents. The number of live (green) and dead (red) cells in each fluorescence image was quantified with NIH ImageJ software. The percentage of dead cells was calculated and compared between groups.

### Statistical analysis

Two way ANOVA test with multiple comparisons was used to determine the statistical difference in experiments with more than two groups. Student's *t-test* was used in experiments involving two groups. Statistical significance was accepted at *p* < 0.05.
